# Editorial: Exercise intervention for prevention and management of type 2 diabetes

**DOI:** 10.3389/fphys.2023.1320101

**Published:** 2023-10-23

**Authors:** Céline Aguer, Smiti Snigdha, Lauren M. Sparks

**Affiliations:** ^1^ Department of Physiology, Faculty of Medicine and Health Sciences, McGill University—Campus Outaouais, Gatineau, QC, Canada; ^2^ Institut du Savoir Montfort, Ottawa, ON, Canada; ^3^ Department of Biochemistry, Microbiology and Immunology, Faculty of Medicine, University of Ottawa, Ottawa, ON, Canada; ^4^ School of Human Kinetics, Faculty of Health Sciences, University of Ottawa, Ottawa, ON, Canada; ^5^ Interdisciplinary School of Health Sciences, Faculty of Health Sciences, University of Ottawa, Ottawa, ON, Canada; ^6^ Translational Research Institute, AdventHealth, Orlando, FL, United States

**Keywords:** exercice training, type 2 diabetes, moderate intensity exercise, high intensity exercise, physical inactivity, extracellular vesicles, sodium-glucose cotransporter 2 inhibitor, mitochondria

More than half a billion people are living with diabetes worldwide, and almost all global cases (96%) are type 2 diabetes (T2D). With that number projected to more than double to 1.3 billion people in the next 30 years, the need for effective pharmacological and non-pharmacological prevention and treatment strategies is unmet and dire. Obesity and physical inactivity (i.e., sedentary lifestyle) are two significant risk factors for the development and progression of T2D. The molecular mechanisms driving the disease onset and progression in distinct peripheral tissues, as well as their cross-talk, remain unclear. In addition to pharmacological treatments such as glucose-lowering medications (e.g., metformin, glucagon-like peptide 1 (GLP1) agonists, sodium-glucose cotransporter-2 (SGLT2) inhibitors, etc.), physical activity and structured exercise (exercise) are well-known therapeutic strategies for both prevention and management of T2D. Comparatively, adherence is high for medication use, while exercise adherence is often low for a variety of reasons such as lack of time, pain, costs, lack of expected results, thus making it difficult to prescribe physical activity alone or in combination with pharmacotherapy to patients with T2D. In recent years, the exercise physiology field has shifted its focus toward finding the type of exercise training that could improve adherence and answer the question of “how little can I do?” For example, replacing longer moderate intensity aerobic exercise bouts with shorter and more intense exercises such as those practiced in high intensity exercise training (HIIT) has yielded promising results. This area of research requires further investigations in larger and more diverse cohorts to reach the goal of prescribing the optimal program for individuals with T2D that will not only manage their symptoms but also promote lifetime adherence.

In this Research Topic, we aimed to shed more light on how decreasing sedentary time and/or increasing structured exercise training—either alone or in combination with diet or pharmacotherapy—aids in the management of T2D at the molecular, cellular, and physiological levels with the ultimate goal of finding new avenues to help individuals with T2D manage their disease. Three original articles and two reviews have been published on this topic by authors around the world. We summarize here the significance of these publications.

The original research by Domazet et al. comprehensively assesses the patterns of physical activity in patients newly diagnosed with T2D to determine whether they achieved the minimal levels of physical activity recommended and to determine the factors influencing active and sedentary behaviours. The Danish Health Authority recommends 60 min per day of moderate to high intensity physical activity. Interestingly, in this cohort of about 800 Danish patients with T2D, while most of them (62%) achieved the recommended level of moderate to intense physical activity per day, they were still sitting almost 10 h a day and walking an average of less than 4,000 steps/day; thus, these patients were still engaging in sedentary behaviour for the majority of their day. These results highlight the popular notion that reducing sedentary time (time spent sitting or lying down) and increasing time spent engaging in any level of physical activity (e.g., walking) is equally as important in managing T2D as participating in structured exercise programs of moderate to vigorous intensity. Furthermore, the manuscript by Domazet et al. also highlights the factors that might influence the development of an unfavorable physical activity lifestyle (i.e., high sedentary time and low moderate-to-vigorous physical activity) such as age, obesity, unemployment, retirement, having co-morbidities associated with T2D, and currently smoking. The authors therefore recommend that future physical activity interventions should target individuals with T2D with these risk factors in particular.

Complementing the study by Domazet et al. *,* the review by Handy and Holloway provides a critical overview of the link between physical inactivity and the development of insulin resistance, independent of overconsumption of energy and/or fat. The authors point out that the development of insulin resistance in the contrasting paradigms of skeletal muscle disuse and overfeeding is likely the result of different and independent mechanisms. Indeed, high calorie consumption and/or lipid overfeeding typically result in accumulation of lipid intermediaries and/or reactive oxygen species (ROS) that interfere with the insulin signaling pathway and lead to peripheral insulin resistance, especially in the skeletal muscle and liver. While lipids can also induce mitochondrial dysfunction through various mechanisms such as inhibition of mitochondrial ADP transport via adenine nucleotide translocase (ANT), the authors highlight that mitochondrial dysfunction and insulin resistance are not always linked. In contrast to overfeeding and lipid overload, skeletal muscle insulin resistance in the context of lack of physical activity and muscle disuse results from decreased synthesis of proteins in the insulin signaling pathway and mitochondrial metabolism. The review also discusses the advantages and disadvantages of different physical activity modalities in improving muscle insulin sensitivity. The authors indicate that although HIIT has gained popularity over the past decade, moderate intensity exercise training appears to be more efficient in improving muscle insulin sensitivity and mitochondrial function. The molecular mechanisms that drive the improved insulin sensitivity in response to chronic exercise include the upregulation of peroxisome proliferator-activated gamma coactivator 1-α (PGC-1α), which is involved in the regulation of the insulin signaling pathway, mitochondrial biogenesis, and neutral lipid synthesis. The emerging importance of increased mitophagy and its role in improved peripheral insulin sensitivity in response to exercise training is also discussed. To summarize, the review by Handy and Holloway elegantly discusses the importance of the interactions between mitochondrial biology, physical activity, and lipid metabolism for the regulation of peripheral insulin sensitivity.

Another mechanism that could explain the positive effect of exercise for patients with T2D is through the release by contracting muscle of extracellular vesicles (EVs) such as exosomes and microvesicles (MVs) that could potentially target local and distant tissues by releasing their cargo. In this context, the original manuscript by Aas et al. explores how the contents of exosomes and MVs are altered in response to muscle contraction in patients with T2D. By using a recognized *in vitro* model of muscle contraction (human primary myotubes treated for 24 h with electrical pulse stimulation (EPS)), the authors described how EPS modifies the contents of exosomes and MVs released by myotubes derived from women with obesity and T2D. While the sizes of exosomes and MVs were not altered by EPS, the miRNA and protein contents of these EVs were modified in response to EPS. Specifically, the authors identified, in exosomes and/or MVs, 4 miRNAs (miR-4433b-3p, miR-92b-5p, miR-320b, miR-1233-5p) and 4 proteins of interest (CNDP2 (cytosolic non-specific dipeptidase), GANAB (Neutral alpha-glucosidase AB), HSPA9 (Stress-70 protein, mitochondrial), and ATP5B (ATP synthase subunit beta, mitochondrial)) that were altered by the EPS treatment and that could play a role in obesity/T2D or exercise training adaptations. The miR that most excited the authors was miR-4433b-3p, which was almost doubled with EPS. MiR-433b-3p is indeed very interesting since it targets CNDP2, which was also almost doubled in exosomes with EPS. CNDP2 regulates the production of LacPhe, a metabolite produced during exercise and recognized as regulating appetite. In summary, Aas et al. manuscript demonstrates the utility of primary human muscle cells as models of human muscle metabolism and exercise. These findings also highlight how muscle contractions alter the content of EVs released by myotubes from women with T2D and opens door to new research that should help identifying the role of EVs in the physiological adaptations to exercise in the context of T2D.

The original article by Valsdottir et al. interrogates the concept of the potential synergistic effects of combined aerobic exercise with caloric restriction on a normal diet or a low carbohydrate/high fat diet (LCHF) on weight loss, glucose tolerance and fat patterning (i.e., distribution of android and gynoid adipose tissues) in middle-aged women with overweight and obesity at risk for developing T2D using a randomized controlled trial (RCT). A total of 57 women were randomized to four groups for a 10-week supervised intervention: 1) normal diet, no exercise (NORM), 2) LCHF, no exercise (LCHF), 3) normal diet plus exercise (NORM-EX) and 4) LCHF diet plus exercise (LCHF-EX). Cardiorespiratory fitness (absolute VO_2_peak) significantly improved in the two EX groups with adherence between 88% and 93%. Similar weight loss was achieved in all four groups ranging from 5.2 kg in the NORM group up to 6.7 kg in the LCHF-EX group. With all groups pooled, weight loss was 6.7%. A significant reduction in the area under curve (AUC) for glucose was observed within all the groups except for the LCHF group. Whereas AUC for insulin was significantly reduced in NORM-EX and LCHF-EX with no substantial changes observed in NORM or LCHF groups. HOMA-IR was only improved in the LCHF-EX group, while Matsuda insulin sensitivity index (ISI) showed a sizable increase in all but the LCHF group. In terms of fat patterning, all groups achieved a robust reduction in android and gynoid fat masses in response to the intervention, with greater reductions in android and the most robust responses observed in the LCHF and LCHF-EX groups, respectively. The key finding was matched weight loss during a 10-week program with diet only, or with a combination of exercise and diet, resulted in improvements exclusively in the exercise groups, in terms of cardiorespiratory fitness and AUC insulin with no distinct impacts on AUC glucose. Collectively, these results emphasize the positive effects and importance of exercise during a weight-loss program and has significant implications for both prevention and management of T2D.

SGLT2 inhibitors, such as canagliflozin, dapagliflozin, empagliflozin, and ertugliflozin, are the recent class of FDA-approved drugs that have proven to be very effective antihyperglycemic agents for adult patients with T2D. More recently, this drug class has also shown reductions in body weight, as well as reductions in adverse cardiovascular events and hospitalizations due to heart failure in individuals with obesity and T2D. As a result, these drugs are highly sought after by patients and physicians alike; however, their potential synergy or use as adjuvant therapy to lifestyle interventions such as diet and exercise is an area of active investigation. Peng et al. conducted a meta-analysis according to Preferred Reporting Items for Systematic Reviews and Metanalyses (PRISMA) guidelines. According to the participant, intervention, comparison, and outcome (PICO) elements, inclusion criteria were based on four factors: 1) types of studies: RCTs or non-RCTs that evaluated the effect of SGLT2 inhibitor intervention on cardiopulmonary endurance; 2) types of participants: patients that were diagnosed with T2DM, chronic heart failure, or obesity (age ≥ 18, both gender); 3) types of interventions: the intervention measures of the experimental group were SGLT-2 inhibitors as single-drug treatment or SGLT2 inhibitors combined with other anti-diabetic agents on the basis of exercise combined with diet intervention; and 4) types of outcome measures: VO_2_peak was taken as the primary outcome, while the respiratory exchange ratio (RER), VE/VCO_2_ slope (minute ventilation/carbon dioxide production), and VAT (ventilatory anaerobic threshold) exercise capacity parameters were taken as the secondary outcomes to evaluate the effect of SGLT2 inhibitors on exercise capacity. The meta-analysis revealed that SGLT2 inhibitors can in fact increase the VO_2_peak and VAT in some populations of individuals with obesity with stable chronic heart failure, and at high risk for cardiovascular disease with or without T2D. In addition, they pointed to improvements in exercise tolerance in individuals with T2D combined with heart failure. However, SGLT2 inhibitors did not impact the exercise parameters of the VE/VCO_2_ slope and RER in similar populations. The current suggested mechanisms for these effects are the ability of SGLT2 inhibitors to elevate hematocrit and erythropoietin to increase oxygen delivery, improve mitochondrial fatty acid oxidation in skeletal muscle, induce weight loss, increase synthesis of ketone bodies, and convert energy metabolism substrate from glucose to fatty acid oxidation for utilization by the heart. These findings are encouraging for the potential impact of SGLT2 inhibitors on certain aspects of exercise capacity and cardiorespiratory fitness. Further evidence-based studies in larger cohorts will be beneficial to determine the effects of SGLT2 inhibitors alone and in combination with exercise on known deficits in exercise tolerance and fitness in individuals with obesity, T2D and heart failure to improve prognosis and reduce the all-cause of mortality risk.

Considerable advancements have been made in the past few decades to improve management and prevention of T2D through the use of pharmacotherapy, as well as non-pharmacologic strategies such as diet and exercise. Despite this significant progress, however, the number of cases of T2D continues to rise, and the quality of life for those individuals living with T2D declines. Low exercise adherence limits its utility and effectiveness as a therapeutic tool. The exercise physiology field continues to address this issue in creative ways through promoting decreased sedentary time and increased low-to-moderate physical activity–such as walking–that is easily maintained throughout life. In addition, the recent push to combine effective pharmacotherapy with exercise is another attractive treatment strategy that holds promise for long-term adherence and care. In terms of prevention, as well as treatment, interrogating the underlying mechanisms that drive both the development and progression or worsening of T2D in a tissue-specific manner continues to be a top priority for clinical, translational, and basic science researchers in the field.

